# Supplementary material for: The Cationic Amphiphilic Drug Hexamethylene Amiloride Eradicates Bulk Breast Cancer Cells and Therapy-Resistant Subpopulations with Similar Efficiencies
Source: Cancers (Basel). 2022 Feb 14;14(4):949. doi: 10.3390/cancers14040949 (PMC8869814; doi:10.3390/cancers14040949)
Supplement: Supplementary file 1 [file cancers-14-00949-s001.zip › cancers-1561611-Supplementary Materials.pdf]

Supplementary Files

The Cationic Amphiphilic Drug Hexamethylene Amiloride Eradicates Bulk Breast Cancer Cells and Therapy-Resistant Subpopulations with Similar Efficiencies

Anastasia L. Berg [1,2], Ashley Rowson-Hodel [1,2], Michelle Hu [1,2], Michael Keeling [1,2], Hao Wu [1,2], Kacey VanderVorst [1,2], Jenny J. Chen [1,2], Jason Hatakeyama [1,2] , Joseph Jilek [1,2], Courtney A. Dreyer [1,2], Madelyn R. Wheeler [1,2], Ai-Ming Yu [1,2], Yuanpei Li [1,2] and Kermit L. Carraway III [1,2,*]

[1] Department of Biochemistry and Molecular Medicine, University of California, Sacramento, CA 95817, USA; alberg@ucdavis.edu (A.L.B.); arhodel@ucdavis.edu (A.R.-H.); mghu@ucdavis.edu (M.H.); mtkeeling@yahoo.com (M.K.); hhwu@ucdavis.edu (H.W.); kvandervorst@ucdavis.edu (K.V.); wxlchen@ucdavis.edu (J.J.C.); jhatake@ucdavis.edu (J.H.); jljilek@pharmacy.arizona.edu (J.J.); cadreyer@ucdavis.edu (C.A.D.); mrkring@ucdavis.edu (M.R.W.); aimyu@ucdavis.edu (A.-M.Y.); lypli@UCDAVIS.EDU (Y.L.)

[2] Davis Comprehensive Cancer Center, University of California Davis School of Medicine, Sacramento, CA, 95817, USA;

Table S1. Characteristics of breast cancer cell lines. Breast and mammary cell lines were previously derived from both tumor and non-tumorigenic tissue types and classified based on cell of origin (basal or luminal) as well as hormone receptor expression status (estrogen receptor [ER], progesterone receptor [PR], and epidermal growth factor receptor 2/human epidermal growth factor receptor 2 [ERBB2/HER2]). Receptor expression is denoted as positive (+), negative (−), or unknown (unk). Cell lines were either purchased from American Type Culture Collection (ATCC) or gifted.

Cell Line	Subtype	ER	PR	ERBB2/HER2	Tissue Type	Source
Human						
MDA-MB-231	Basal	-	-	-	Metastatic Adenocarcinoma	ATCC #HTB-26
MCF7	Luminal	+	+	-	Metastatic Adenocarcinoma	ATCC #HTB-22
SKBR3	Luminal	-	-	+	Adenocarcinoma	ATCC #HTB-30
T47D	Luminal	+	+	-	Invasive Ductal Carcinoma	ATCCC #HTB-133
MCF10A	Basal	-	-	-	Non-tumorigenic	ATCC #CRL-10317
HMEC4	Luminal	unk	unk	unk	Non-tumorigenic	Gifted by K. Rao
Mouse						
NDL	Luminal	-	-	+	Invasive Ductal Carcinoma	Primary tumors
Met-1	Luminal	-	unk	unk	Metastatic Adenocarcinoma	Gifted by A. Borowsky
4T1	Luminal and Basal	-	-	-	Metastatic Adenocarcinoma	ATCC #CRL-2539

nMuMG	Luminal	-	-	unk	Non-tumorigenic	ATCC #CRL-1636

Table S2. Characteristics of non-mammary cancer cell lines. (**A**) Human tumor-derived cell lines representing an array of non-mammary origin cancers are listed with descriptions of tissue origin and source. All cell lines were purchased from ATCC, and catalog numbers are included.

Cell Line	Tissue Type	Source
A549	Lung Adenocarcinoma	ATCC #CCL-185
Du145	Prostate Carcinoma	ATCC #HTB-81
HepG2	Hepatocellular Carcinoma	ATCC #HB-8065
J82	Transitional Cell Carcinoma (Bladder)	ATCC #HTB-1
LS174T	Colon Adenocarcinoma	ATCC #CL-188
Panc-1	Pancreatic Ductal Cell Carcinoma	ATCC #CRL-1469
T98G	Glioblastoma	ATCC #CRL-1690

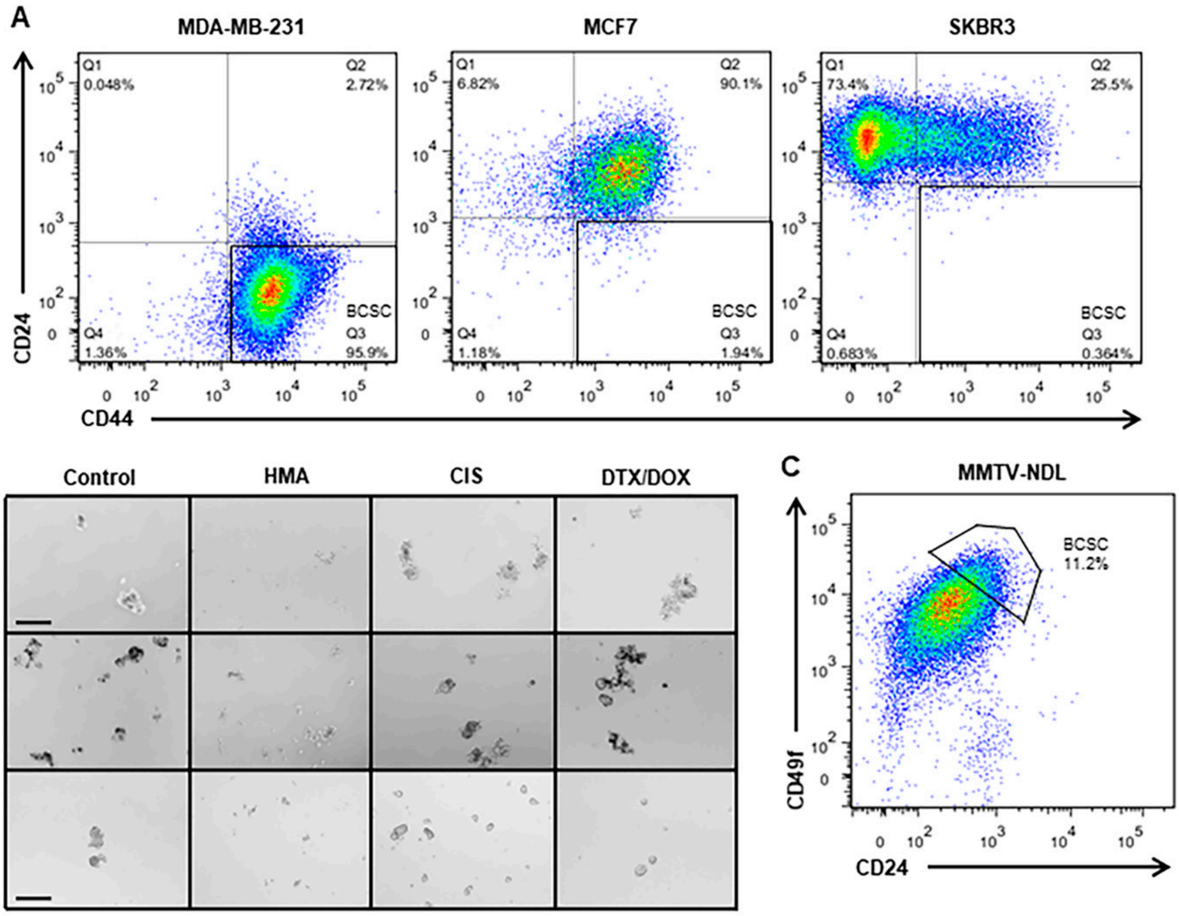

Figure S1. The BCSC subpopulation is susceptible to HMA-induced death. (**A**) FACS dot plot showing cell surface markers used to isolate human BCSCs ($CD44^{+}/CD24^{low}$, Q3). (**B**) Representative images of 7-day spheres grown from $CD44^{+}/CD24^{-}$ BCSC-enriched human breast cancer cells (MDA-MB-231 and MCF7) and mouse mammary tumor cells (MMTV-NDL) following treatment with 40 μM HMA, 40 μM cisplatin (CIS), or a combination of apoptosis inducing agents (170 nM doxorubicin (DOX) and 50 nM docetaxel (DTX)). Scale bar = 200 μm. **(C)** FACS dot plot showing cell surface markers used to isolate mouse primary tumor-derived BCSCs ($CD24^{high}/CD49f^{high}/Lin^{-}$).

.

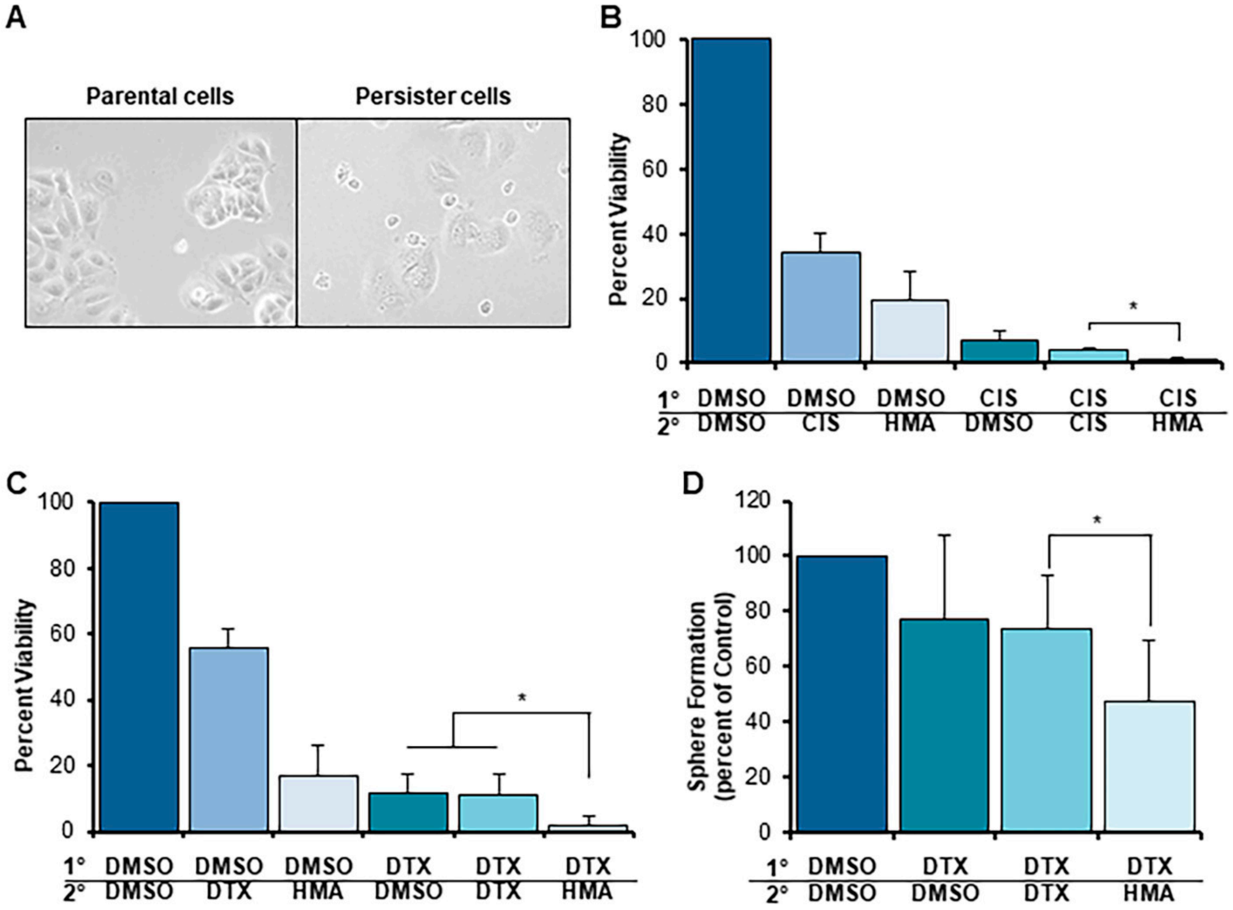

Figure S2. Chemoresistant cells are susceptible to HMA. (**A**) Representative images of parental and persister MCF7 cells are displayed. (**B**) Met-1 mouse mammary cancer cells were exposed to vehicle only control (DMSO) or 100 μM CIS for 48 h (primary treatment [1°]) followed by a subsequent treatment of vehicle, 40μM HMA, or a second dose of CIS (100 μM) for 24 h (secondary treatment [2°]). Viability was determined by trypan blue exclusion assay. (**C,D**) Met-1 mouse mammary cells were administered 1° treatments of vehicle or 500 nM DTX and 2° treatments of vehicle, 40μM HMA, or 500 nM DTX as in (**B**), and cell viability was quantified (**C**). Equal numbers of post-treatment cells were plated in serum-free, low adherent conditions to enrich for the chemotherapy-insensitive BCSC population, and sphere count was determined after 7 days in culture (**D**). Data in (**B–D**) are presented as averages of six replicate experiments ± SD and are compared to the vehicle only control (DMSO-DMSO). $p < 0.05$ *.

.

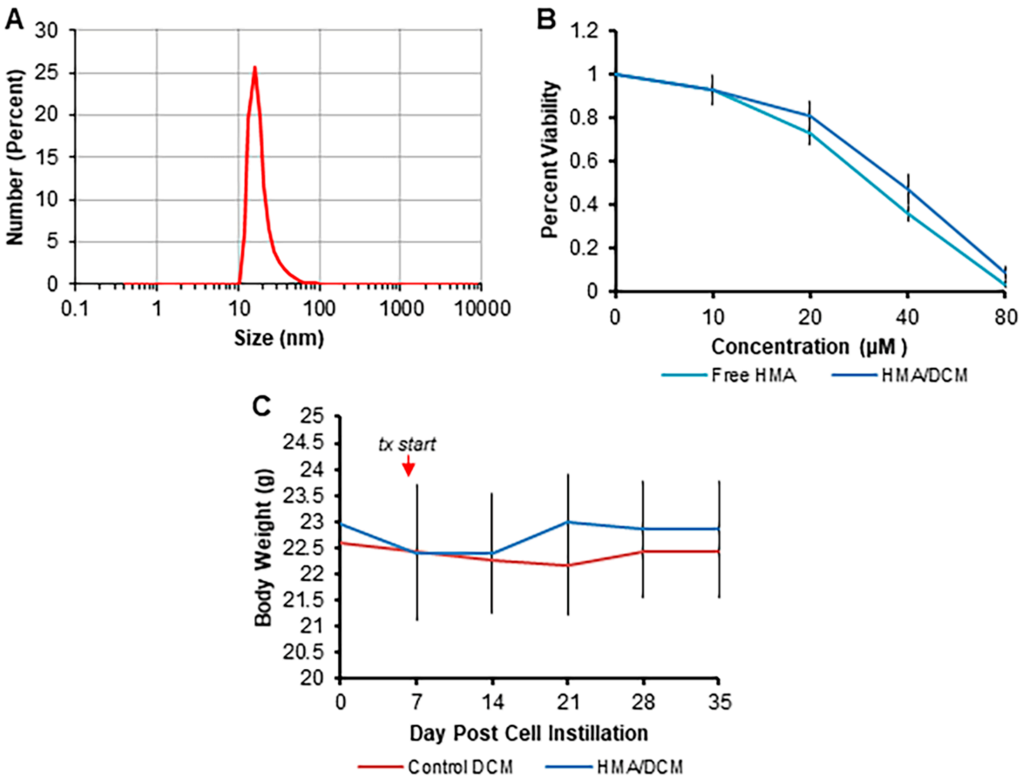

Figure S3. Preparation and characterization of HMA-loaded DCM nanoparticles. DCMs were formed by oxidation of thiol groups to disulfide bond within the micelle core following self-assembly of a thiolated linear-dendritic polymer (telodendrimer) composed of polyethylene glycol (PEG) attached to a dendritic oligomer of cholic acids (CAs) through a poly(lysine (L)-cysteine (Cys)-Ebes) backbone ([PEG^{5k}-Cys_4-L_8-CA_8] indicating the length of PEG) [38]. Micelle crosslinking enhances structural stability *in vivo* and slows drug release for improved duration in systemic circulation, and DCMs demonstrate tumor site accumulation due to the enhanced permeability and retention effect [38]. (**A**) The size of HMA-loaded DCMs was measured by a dynamic light scattering (DLS) instrument (Microtrac. Osaka, JPN). The final concentration of polymers was kept at 20 mg/mL. HMA loading was 5 mg/mL (loading rate was 89.5%). Size distribution of loaded DCMs is displayed (average particle size was 19.8 nm). (**B**) NDL tumor cells were treated for 24 h with free HMA or DCM/HMA, and viability was assessed by MTT assay. Data is presented as the average of 3 biological replicate experiments ± SEM. **(C)** Average body weight of FVB/NJ mice injected with NDL cells via tail vein over the course of intravenous DCM/HMA treatment.
